# Changes in Physical Activity Involvement and Attitude to Physical Activity in a 16-Year Follow-Up Study among the Elderly

**DOI:** 10.4061/2010/174290

**Published:** 2010-07-15

**Authors:** Mäkilä Päivi, Hirvensalo Mirja, Parkatti Terttu

**Affiliations:** ^1^Well-Being Services, Turku University of Applied Sciences, Ruiskatu 8, 20720 Turku, Finland; ^2^Faculty of Sport and Health Sciences, University of Jyväskylä, P.O.Box 35 (L), 40014 Jyväskylä, Finland

## Abstract

We studied changes of physical activity among noninstitutionalized 65 years and older persons over a sixteen-year follow-up period. The focus of our interest was on changes in involvement, frequency, intensity, and various modes of physical activity. Furthermore, we studied changes in perceived importance, motives for, and obstacles to participation in physical activity. The results showed that the proportion of those reporting less frequent and intensive activities increased. Men were more active than women over the follow-up time (in 1988 *P* = .015, in 1996 *P* = .007, in 2004 *P* = .001). The biggest difference at the end of the followup between men and women was found in participation in supervised exercise classes (39% and 14%, resp.). Most popular forms of physical activity were walking and calisthenics at home. Men undertook more modes of physical activity than women. The importance of physical activity declined during the followup in both gender groups but more among women than men. The most common obstacles to physical activity were poor health and lack of interest. The promotion of health maintained it's place as the most important reason for physical activity over the follow-up period.

## 1. Introduction

Physical activity has an important role in the well-being of aging people. its importance for maintaining physical capacity and health during a person's lifetime has been well documented [1–7]. Furthermore, physical activity contributes to mental well-being offering, for example, possibilities to maintain social networks [8, 9].

The modes, regularity, and intensity of physical activity change with increasing age, so that the total time spent in physical activity and, particularly, the regularity and intensity of the activity clearly decline after 75–80 years of age [10–12]. Health promoting physical activity seems to decrease during the lifetime, but there are big differences between individuals. Although some are engaged in optimal levels of physical activity, others develop lifestyles that are generally sedentary [13]. Furthermore, differences appear between gender groups. It has been explained by gender-roles that physical activity and sports has been men's domain, and women were presumed to get all the exercise they needed in their domestic chores [14]. 

To be able to develop physical activity interventions for older people, it is important to know how different aspects of physical activity change with increasing age. What happens to attitude, motives, and barriers to physical activity. Although the number of studies on older people's physical activity is growing rapidly, longitudinal studies of physical activity of older people are still very rare.

Different aspects of physical activity have been covered in several international longitudinal studies [15–17] but involvement, types frequency and intensity are rarely followed over time with same people. Nottingham longitudinal study [15] has shown declining changes in physical activity outdoor with leisure activities declining the most. On the other hand, the number of those who use more time for walking increased [18–20]. 

Health refers often to the most important motive for physical activity among older people [21–24]. According to a review article by Schutzer and Graves [25], the level of one's knowledge about physical activity and its importance does not necessarily translate into adhering to a long-term physical activity regimen. Over time, perceived feelings of enjoyment and satisfaction have appeared to better predict higher levels of adherence [26, 27]. The most often mentioned obstacles to physical activity among older people are poor health, lack of time, the effects of slowing down due to ageing, reduced strength, and endurance and the weakening of pulmonary function, functional limitations and environmental factors [24, 28, 29]. Although motives for and obstacles to physical activity have been studied before, there is a lack of studies concerning the change of older people's attitudes to physical activity over time. In addition, to our knowledge any studies about the perceived importance of physical activity among the elderly have not yet been published. 

In this study, the concept of physical activity is defined according to Caspersen et al. [30] as a body movement that is produced by contraction of skeletal muscles and increases energy expenditure. The aim of this study is to describe and examine the change of physical activity participation in a representative sample of elderly people over a sixteen-year follow-up period. The changes in involvement during follow-up were studied in various modes of physical activity. Furthermore, we studied changes in perceived importance, and motives for and obstacles to participation among people born between 1914 and 1923, who were living in their own homes during the baseline interview in 1988.

## 2. Materials and Methods

### 2.1. Study Design and Participants

**Figure 1:**
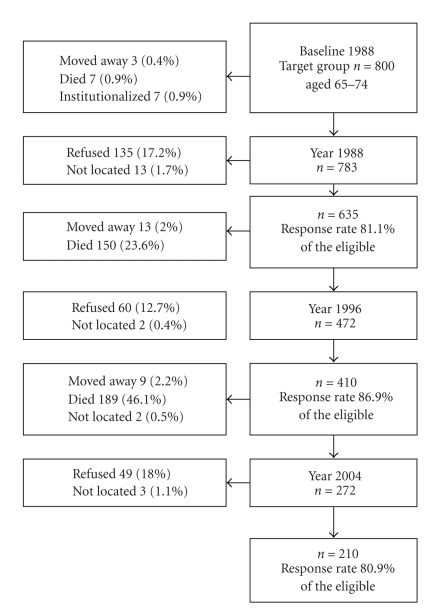
Participation in the study.

The data reported in this article is part of a larger, regionally representative 16-year follow-up study of the Evergreen project consisting of elderly people's health, functional capacity, and well-being. The study was carried out among residents aged 65 and over in a medium-sized town, Jyväskylä, in central Finland. The total population of Jyväskylä at the time of the baseline survey, in 1988, was 65 000, of whom 11.6%  (*n* = 7600) were 65 and over. At the baseline, the target population comprised 1600 residents of Jyväskylä born in 1904–23. Two representative random samples of 800 respondents, men and women aged 65 to 74 and 75 to 84, were drawn from the population register. Approximately 80% of them (*n* = 1224) took part in the baseline interview. At baseline, only those who were living in their own homes were included in the study. The final sample size was 1185. The data from the younger cohort, born 1914–23 is used in this article because of the small number of survivors in the older cohort in 2004. In 1988, 635 persons from the younger age cohort, representing 81% of the target group (*n* = 800), took part in the baseline interview. The study plan has been described in more detail elsewhere [31]. The participation and reasons for drop-outs of the younger cohort in the study are presented in Figure 1. Altogether 28% of the participants from the baseline interview took part in the interview in 2004. The analysis of the change in physical activity during the follow-up period included also the data of those who had died during the follow-up. 

The first follow-up study in 1996 consisted of those who had been interviewed in 1988 and still lived in the same town. The follow-up study in 2004 consisted of those who participated in 1988 and in 1996 as well as 21 persons, who had participated in 1988, but not in 1996. In the both follow-up interviews, we also interviewed those, who had been institutionalized (in 1996 3%, *n* = 13, in 2004 10%, 21) during the follow-up period.

### 2.2. Data Collection

The data was collected by interviews using a structured questionnaire. Trained interviewer visited residents in their homes on two occasions. The interviews lasted approximately two hours each including, among other questions, 19 questions about forms and intensity as well as motives, importance, and barriers of physical activity. First, we assessed physical activity according to both frequency and intensity using a question with seven categories that ranged from doing the necessary household chores to competitive sports. By the time of baseline study in 1988, this question was considered a valid and reliable measurement for physical activity [32]. The categories were (1) moving only for necessary chores, (2) walking or other outdoor activities 1-2 times a week, (3) walking or other outdoor activities several times a week, (4) exercising 1-2 times a week to the point of perspiring and heavy breathing, (5) exercising more than 1-2 times a week to the point of perspiring and heavy breathing, (6) exercising for fitness several times a week to the point of perspiring and heavy breathing, and (7) keeping fit through regular heavy physical activity or competitive sport. We removed the “not relevant” category from this variable and combined competitive sports with the category that included doing active, keep-fit activities, because both of these categories included only a few responses. The scale was modified from Grimby [32].

For further knowledge of physical activity of older people, we studied modes of physical activity by asking the respondents to identify the activities, in which they took part in order to maintain their health and physical fitness. The modes of physical activity listed in the questionnaire were walking for fitness, callisthenics at home, swimming, cycling, cross-country skiing, dancing, supervised physical activities classes, and other. We measured the frequency of different modes of physical activity using a 6-point scale: (1) almost daily, (2) 2-3 times a week, (3) weekly, (4) 2-3 times per month, (5) once a month or more rarely, and (6) nonparticipation. The importance of physical activity was assessed using a five-point scale question “The aim of the next question is to clarify the importance of physical activity to you” (1 = *I* consider physical activity very important, 2 = *I* consider physical activity somewhat important, for example, for health, 3 = difficult to say, 4 = physical activity is not very important to me, 5 = *I* consider physical activity as useless and wasting of time). Regarding barriers to physical activity, we asked participants to mention three factors in order of importance. We classified the responses into five groups: lack of time (e.g., household activities, other hobbies), lack of interest, poor health (e.g., fatigue, poor fitness, other illnesses, psychological factors), social factors (e.g., not having a friend, lack of social support), and other obstacles (e.g., fear of exercising outside, lack of knowledge, physical discomfort, being too old). The classified variable was then used in the follow-up questionnaires.

### 2.3. Statistical Methods

Descriptive statistics, percentage, and frequency were used to evaluate the prevalence of physical activity, its importance, reasons and barriers. The change in physical activity and its importance over the 8-year follow-up period (two points of time) was analysed using the McNemar test. During the 16-year follow-up period (three points of time), the Cochran Q-test was used. The choice of statistical test was based on the scale of measurement. Due to insufficient number of responses in some categories, reclassification was done where warranted by the contents. To compare men and women, and participants who were interviewed in 2004 with those who had died during the follow-up period, *χ* ^2^-test was used. Statistical analyses were executed by using the SPSS 15.0 software package.

## 3. Results

Results show that at baseline 38%  (*n* = 241) of the participants were male and 62%  (*n* = 394) were female. The mean age of participants was 69 years in 1988, 76 years in1996, and 84 years in 2004. During the sixteen-year follow-up period, 54%  (*n* = 346) people died and 4%  (*n* = 25) moved away from Jyväskylä. 

Table 1 shows the self-reported physical activity at baseline and in different points of the follow-up. The proportion of those reporting less frequent and intensive activity increased as participation in more intensive physical activity declined. Men were more active than women in 1988 (*χ* ^2^ (5) = 14, *P* = .015), in 1996 (*χ* ^2^(5) = 16, *P* = .007), and in 2004 (*χ* ^2^(5) = 16, *P* < .001). Those men and women, who died during the follow-up period, had been physically less active at baseline than those, who participated in all of the assessments in 1988, 1996, and 2004 (*P* < .001 (Not in table)). 

Most popular forms of physical activity among participants were walking and calisthenics at home. 

 Proportions of persons reporting physical activity decreased in both gender groups over the follow-up time, with the only exception being increased participation of men in supervised exercise classes (*P* < .001) and callisthenics at home which was, however, not statistically significant (Table 2). About 39% of men undertook supervised exercise classes at the end of 16-year follow-up while the prevalence was only 14,6% among women. The number of those performing callisthenics at home during the 8- and 16-year follow-up studies increased among men, whereas among women it remained the same. At the end of the 16-year follow-up study in 2004, sixty-eight percent of women and sixty percent of men performed callisthenics at home at least 2-3 times per week. Participation in swimming, cycling, cross-country skiing, and dancing was less common. However, men undertook more modes of physical activity than women. 

Most of the participants considered physical activity to be at least somewhat important (Table 3). However, during the 8- and 16-year follow-up studies there was a statistically significant decline (*P* = .008 and *P* = .002, resp.) in the proportion of women who considered physical activity to be very important. No significant change among men was observed. 

The most common obstacles to physical activity reported at baseline in 1988 by both gender groups included poor health (19%), lack of interest (6%), lack of time (2%), and other reasons (2%). About half (46,2%) of the respondent did not name any obstacle. During the 8-year and 16-year follow-up studies, the percentage of people, who faced some barrier to physical activity increased. In 2004, the obstacles most often mentioned in order of importance among men and women were poor health and lack of interest. 

The reasons for physical activity were similar in 1988, 1996, and 2004. The most important reason given at every point of measurement was the promotion of good health (58,9%, 70,4%, 73,9%, resp.). Participants also mentioned social reasons (friends, togetherness), and the satisfaction derived from physical activity as motivating factors. 

## 4. Discussion

In this study, physical activity declined both in the 8- and 16-year follow-up studies, though less among men than women. However, almost 70% of the 81–90-year-old men and over 40% of women reported walking several times a week or even at times engaging in more intensive levels of physical activity. Men took part actively in supervised exercise classes and they reported a wider range of activities than women at the end of the study in 2004. Although a considerable proportion of elderly people were physically active, over half of women and one third of men reported low levels of activity. Also, the proportion of those women, who considered physical activity to be very important, declined significantly during the follow-up periods. In contrast, most men still considered physical activity to be very important at the end of the follow-up. 

During the first part of the follow-up, in the 8-year follow-up study, physical activity remained at almost the same level in men but decreased in women. The results are in line with the survey by Gauthier and Smeeding [31], who noticed that men devoted more time to physical activity than women, and that physical activity among men even increased until 74 years of age. However, the decline in levels of physical activity among both men and women during 16-year follow-up study may be related to frailty and living in a more dependent environment. Participants also reported more often than earlier that they were too old to be physically active. On the other hand, during the 8-year and 16-year follow-up studies, participation in supervised exercise classes and callisthenics at home increased among men, whereas among women they remained the same or slightly decreased. The popularity of performing callisthenics at home can be explained by the fact that, when other modes of physical activity decline, it is easy to carry out in a familiar environment within a suitable amount of time, and in a manner that can easily be adjusted to accommodate occasional health and functional limitations. Participating in a supervised exercise classes provided an opportunity for social interaction, which was, besides health reasons, the most often mentioned reason for physical activity. For example, for men, war veterans' exercise groups provide opportunities to meet others with similar life experiences, and, at the same time, to look after their health.

One reason for high level of participating supervised exercise classes may be the well-organized community-based exercise programs for different age groups including great number of supervised exercise classes for over 65-year-old people in the town, where the study was executed. 

During the follow-up time, perceived importance of physical activity declined among women. In women still living in the community, this pattern may reflect the time pressures they face in their multiple roles at this life stage and in earlier life. Long working hours and responsibilities at home may have prevented women from performing physical activity in earlier life, thus hindering their efforts to develop a proper physical activity routine in later life. 

In this study, poor health was the most often mentioned obstacle to physical activity. These results are in line with some previous studies [24, 32], which show that poor health and pain, fear of falling, and being injured represent the obstacles to physical activity that older people mention most frequently. Newson and Kemps [33] have also reported health concerns as a reason for exercising and medical problems as a reason for not exercising among older women. While health status seems to be both motive for and obstacle to physical activity, health care personnel play an important role in delivering information about benefits of physical activity or even giving a physical activity prescription [34] to the older clients, as they meet most of the older population in connection of health check-ups and treatments of diseases. 

In addition to health status, earlier studies mention weather conditions [24], lack of facilities and lack of a proper environment for physical activity, as well as the shortage of physical activity-specific knowledge [33], as factors that prevent people from being physically active. Investments by society, such as a safe environment and adequate supervision, counselling, and more supervised training groups would ensure sufficient possibilities for physical activity among older people.

The value of the current descriptive study is in its long follow-up time, which made it possible to investigate changes in adherence concerning physical activity and levels of involvement in physical activity over the course of 16 years among the same group of elderly people. The sample represents the normal adult population. The same procedure in data collection was used in all follow-ups, which may also cause some problems during the study. New methods are developed during the follow-up time, but to be able to follow the change researchers are tight to use the methods, which have been chosen at the baseline. 

The collection of physical activity information using postal questionnaire has been criticized and includes some well-known limitations [35]. In population studies, it is, however, a largely used method [36]. In this study, interview on the basis of a structured questionnaire gives some extra value, especially, when it is a question of older people as a target group. The interviewer can obtain any required additional information using follow-up questions. 

A good quality of life is an important aim at the end of one's life, and the importance of physical activity for promoting physical, psychological, and social health is generally understood. While older people who keep up their functional and mobile capacity maintain the autonomy [37–40], our findings about the declining importance of physical activity during the course of time among older women and the concurrent decrease in their level of physical activity require further attention.

## Figures and Tables

**Table 1:** Physical activity (frequency and intensity) among men and women aged 64–74 years at baseline and in 8- and 16-year follow-up studies, (%, *P*).

Physical activity	All	8-year follow-up			16-year follow-up			
	1988	1988	1996	McNemar test^a^, *P*	1988	1996	2004	Cochran Q-test^b^, *P*
Men	*n* = 237	*n* = 144	*n* = 144		*n* = 67	*n* = 67	*n* = 67	

Necessary chores	18.1	9.7	19.5		7.6	10.6	22.5	
Walking 1-2 times/week	10.5	13.2	10.4	.170	12.1	10.6	9.4	**.030**
Walking several times/week	42.6	45.8	45.8		39.4	47.0	32.8	
1-2 times/week to the point of perspiring and heavy breathing	8.4	9.0	6.3		9.1	6.1	14.1	
Several times/week to the point of perspiring and heavy breathing	16.5	18.8	15.3		28.8	19.7	17.2	
Keep-fit training or competitive sport several times a week	3.8	3.5	2.8		3.0	6.1	3.1	

	100.0	100.0	100.0		100.0	100.0	100.0	

Women	*n* = 390	*n* = 255	*n* = 255		*n* = 132	*n* = 132	*n* = 132	

Necessary chores	20.3	15.3	27.9		15.4	22.7	37.9	
Walking 1-2 times/week	16.2	16.1	14.5	**.002**	16.2	12.9	18.9	**.001**
Walking several times/week	45.6	45.5	47.5		42.3	53.0	34.8	
1-2 times/week to the point of perspiring and heavy breathing	56	5.5	2.4		6.2	3.0	4.5	
Several times/week to the point of perspiring and heavy breathing	11.3	16.1	6.7		18.5	6.8	3.8	
Keep-fit training or competitive sport several times a week	1.0	1.6	1.2		1.5	1.5	—	

	100.0	100.0	100.0		100.0	100.0	100.0	

^a^Physical activity is divided into two categories (1 = only in necessary chores or walking 1-2 times/week and 2 = walking at least several times/week) because of the few number of responses in some categories.

**Table 2:** Modes of physical activity among men and women born in 1914–1923 aged 65–74 years at baseline and the group followed over 16 years (%, *P*).

Modes of physical activity	Follow-up group				
	Baseline 1988	1988	1996	2004	Cochran Q-test, *P*
Men	*n* = 234–237	*n* = 54–59	*n* = 54–59	*n* = 54–59	

Walking for fitness^a^	77.6	91.6	88.1	81.3	.047
Calisthenics at home^a^	44.3	48.3	55.1	60.3	.055
Swimming^b^	23.6	32.2	27.1	22.0	.276
Cycling^b^	33.3	39.0	33.9	18.6	.004
Cross-country skiing^b^	27.1	48.3	25.9	10.3	**<.001**
Dancing^b^	11.0	11.9	13.6	11.9	.913
Supervised physical training^b^	9.9	11.9	13.6	39.0	**<.001**
Other^b^	8.0	3.7	5.6	7.4	.717

Women	*n* = 389–391	*n* = 92–104	*n* = 92–104	*n* = 92–104	

Walking for fitness^a^	79.1	82.7	81.7	73.1	.271
Calisthenics at home^a^	60.1	63.1	66.0	68.0	.822
Swimming^b^	19.7	24.0	10.6	6.7	**<.001**
Cycling^b^	19.8	29.1	12.6	1.0	**<.001**
Cross-country skiing^b^	8.5	13.6	1.9	0.0	**<.001**
Dancing^b^	5.4	4.9	4.9	1.9	.325
Supervised physical training^b^	13.4	21.4	15.5	14.6	.239
Other^b^	1.3	2.2	4.3	0.0	.135

^a^Walking for fitness and calisthenics at home at least 2-3 times a week. ^b^Other forms of physical exercise at least once a month.

**Table 3:** The importance of physical activity among men and women aged 65–74 years at baseline and in 8- and 16-year follow-up studies, (%, *P*).

		Follow-up groups						
Importance of physical activity	Baseline 1988	8-year follow-up			16-year follow-up			
		1988	1996		1988	1996	2004	
Men	*n* = 232	*n* = 139	*n* = 139	McNemar test^a^, *P*	*n* = 58	*n* = 58	*n* = 58	Cochran Q-test^a^, *P*

Very important	69.0	70.5	66.9	,575	75.9	75.9	63.8	,163
Somewhat important	27.7	28.1	27.3		24.1	22.4	32.8	
Don't know	0.9	0.7	4.3		0.0	1.7	0.0	
Not very important	0.4	0.7	1.4		0.0	0.0	3.4	
Total	100.0	100.0	100.0		100.0	100.0	100.0	

Women	*n* = 379	*n* = 247	*n* = 247		*n* = 102	*n* = 102	*n* = 102	

Very important	67.3	69.2	56.7	**,008**	73.5	57.8	58.8	**,002**
Somewhat important	31.7	30.0	38.5		25.5	42.2	39.2	
Don't know	0.4	0.4	3.6		1.0	0.0	2.0	
Not very important	0.8	0.4	1.2		0.0	0.0	0.0	
Total	100.0	100.0	100.0		100.0	100.0	100.0	

^a^The importance of physical activity is divided into two categories (1 = very important 2 = somewhat important, don't know, and not very important) because of the few number of responses in some categories.
